# Dexamethasone Improves Heat Stroke-Induced Multiorgan Dysfunction and Damage in Rats

**DOI:** 10.3390/ijms151121299

**Published:** 2014-11-18

**Authors:** Chia-Chyuan Liu, Mei-Fen Shih, Yi-Szu Wen, Ying-Hsiu Lai, Tsai-Hsiu Yang

**Affiliations:** 1 Department and Institute of Cosmetic Science, Chia-Nan University of Pharmacy and Science, Tainan 717, Taiwan; E-Mail: ccliu@mail.cnu.edu.tw; 2 Department of Pharmacy, Chia-Nan University of Pharmacy and Science, Tainan 717, Taiwan; E-Mail: meifenshih@mail.cnu.edu.tw; 3 Department of Emergency medicine, Taipei Veterans General Hospital, Taipei 112, Taiwan; E-Mail: yswen@vghtpe.gov.tw; 4 Department of Medical Research and Education, Taipei Veterans General Hospital, Taipei 112, Taiwan; E-Mail: d49405004@gmail.com; 5 Department of Health and Nutrition, Chia-Nan University of Pharmacy and Science, Tainan 717, Taiwan

**Keywords:** heat stroke, multiorgan dysfunction, dexamethasone, hypercoagulable state, cytokines

## Abstract

Dexamethasone (DXM) is known as an immunosuppressive drug used for inflammation control. In the present study, we attempted to examine whether DXM administration could attenuate the hypercoagulable state and the overproduction of pro-inflammatory cytokines, improve arterial hypotension, cerebral ischemia and damage, and vital organ failure in a rat model of heat stroke. The results indicated that all the rats suffering from heat stroke showed high serum levels of tumor necrosis factor-α (TNF-α) and interleukin-1β (IL-1β), accompanied with increased prothrombin time, activated partial thromboplastin time and D-D dimer, and decreased protein C. During the induction period of heat stroke, plasma levels of blood urea nitrogen (BUN), creatinine, glutamic oxaloacetic transaminase (SGOT), glutamic pyruvic transaminase (SGPT), and alkaline phosphatase (ALP), were consistently increased. High striatal levels of glycerol, glutamate, and lactate/pyruvate were simultaneously detected. On the contrary, the mean arterial pressure, plasma levels of interleukin-10 (IL-10), and local cerebral blood flow at the striatum were all decreased. Importantly, intravenous administration of DXM substantially ameliorated the circulatory dysfunction, systematic inflammation, hypercoagulable state, cerebral ischemia and damage during the induction period of heat stroke. These findings demonstrated that DXM may be an alternative therapy that can ameliorate heat stroke victims by attenuating activated coagulation, systemic inflammation, and vital organ ischemia/injury during heat stroke.

## 1. Introduction

A clinical diagnosis of heat stroke suggests that body hyperthermia (over 42 °C) associated with a systemic inflammatory response leads to multiple organ dysfunction, in particular, neurological abnormalities after exposure to high temperature [1,2]. Several lines of evidence indicate that animals share with humans almost the same heat stroke syndromes [3,4]. In rodents, heat stress leads to arterial hypotension, hyperpyrexia, and hypercoagulable state, and excessive activated inflammation may contribute to multiple organ failure (including cerebral, hepatic and renal ischemia, injury, and dysfunction) in heat stroke [3,4,5,6].

Steroidal anti-inflammatory drugs have been shown to decrease the generation of leukotrienes and prostaglandins by inhibiting the secretion of phospholipase A2 and the release of arachidonic acid [7]. Glucocorticoids (GCs) are known to be potent inhibitors of cytokine production and to exert a protective effect against lipopolysaccharide-induced death [7]. In addition, GCs has been shown to be of benefit in the treatment of human and animal-spinal cord injury or cerebral ischemia [8,9]. Our previous results revealed that systemic pretreatment with exogenous GCs, such as DXM, before exposure to heat stress, but not immediate treatment at onset of heat stroke, could increase the survival time via reduction of serum interleukin-1β (IL-1β) in rat heat stroke [10]. However, there are fewer studies showing the immediate treatment with DXM at the onset of heat stroke [10], and it will be more meaningful if survival prolongation after heat stroke attacks is demonstrated. After all, it is not practical to give pretreatment in clinical practice. Meanwhile, there is less attention to evaluate effects of DXM on heat stroke-induced pathophysiological changes, especially for the hypercoagulable state, various cytokines levels, and multiple organ dysfunction. The objective of this study was to observe firstly whether immediate treatment with different doses of DXM has efficacy to elongate survival time, and improve heat stroke-induced circulatory shock, cerebral ischemia and damage in rats. Furthermore, we also investigated whether the ameliorative effects of acute treatment with DXM were associated with inhibition of the hypercoagulable state, multiple organ dysfunction, and changes of systemic cytokines levels after heat stroke induction.

## 2. Results and Discussion

### 2.1. DXM Improves Survival during Heat Stroke in a Dose-Dependent Manner

We see from Table 1 that in anesthetized rats treated with normal saline (0.9% NaCl solution) 70 min after the onset of heat exposure (Ta = 43 °C) followed by room temperature (Ta = 24 °C) exposure, the value for survival time is found to be 24 ± 3 min (*n* = 8). However, immediate treatment with DXM 4, 6, and 8 mg/kg b.w. (i.v.) at the onset of heat stroke increases the survival time in a dose-dependent manner to a new value of 104 ± 9, 204 ± 25, and 268 ± 27 min, respectively.

**Table 1:** Effects of heat exposure (HE; ambient temperature of Ta = 43 °C for 70 min) on survival time values in different groups of rats.

Treatment Groups	Survival Time (min)
1. Normal saline-treated (1 mL/kg, iv) normothermic control rats	>480
2. Normal saline-treated (1 mL/kg, iv) heat stroke rats	24 ± 3 ^†,‡^
3. Dexamethasone (4 mg/kg, iv)-treated heat stroke rats	104 ± 9 *^,‡^
4. Dexamethasone (6 mg/kg, iv)-treated heat stroke rats	204 ± 25 *^,†^
5. Dexamethasone (8 mg/kg, iv)-treated heat stroke rats	268 ± 27 *^,†,‡^

Values are the means ± S.E. of 8 rats per group. Groups 2–5 exposed to 43 °C had heat exposure withdrawn at the onset of heatstroke. * *p* < 0.05 in comparison with group 2; ^†^ *p* < 0.05 in comparison with group 3; ^‡^ *p* < 0.05 in comparison with group 4. (one-way ANOVA, followed by Duncan’s test). Group 1 was killed about 480 min at the end of the experiments with an overdose of urethane.

### 2.2. DXM Ameliorates Arterial Hypotension, Cerebral Ischemia and Damage during Heat Stroke

**Figure 1:**
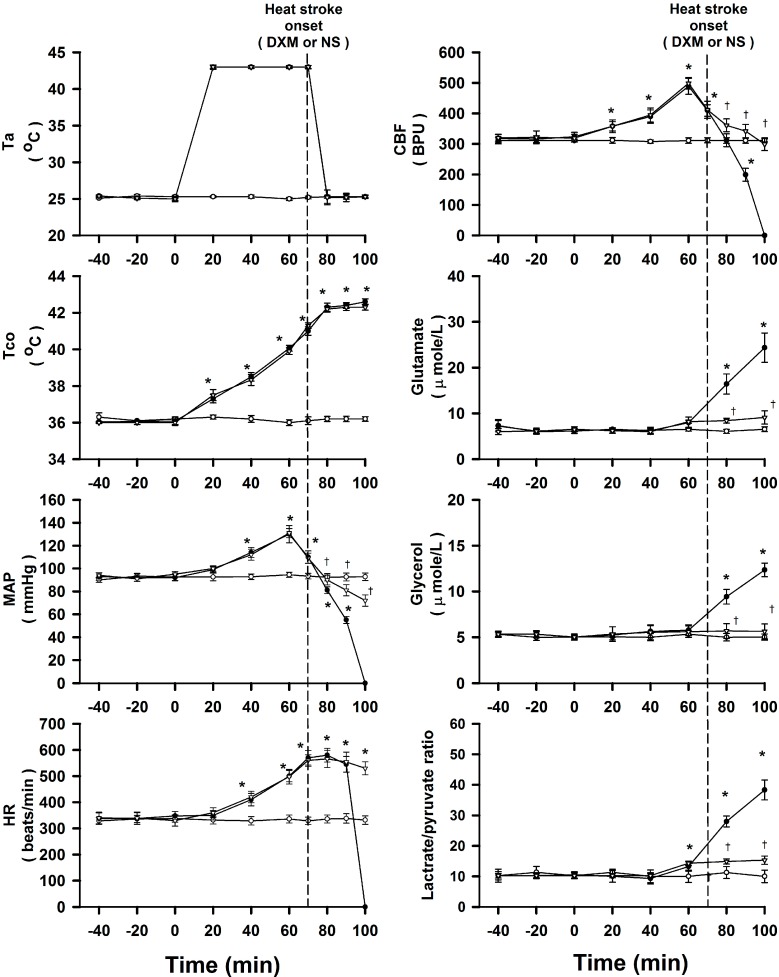
Effects of heat exposure (43 °C) on colonic temperature (Tco), MAP, heart rate (HR), CBF and the extracellular concentrations of glutamate, glycerol, and lactate/pyruvate ratio of the corpus striatum in normothermic control rats (open circles), 0.9% NaCl solution-treated (filled circles, 8 mk/kg b.w., i.v.) or DXM-treated rats (open triangles). The dotted line indicates time of heat stroke onset and drug injection. * *p <* 0.05, compared with normothermic control rats (ANOVA followed by Duncan’s test); ^†^ *p <* 0.05, compared with saline-treated rats (ANOVA followed by Duncan’s test).

As shown in Figure 1, fifteen minutes after the onset of heat stroke in normal saline (0.9% NaCl solution) treated group, all the values of mean arterial pressure (MAP) and cerebral blood flow (CBF) were significantly decreased as compared with those of normothermic controls. On the other hand, the values of extracellular concentrations of glutamate, glycerol, and lactate/pyruvate ratio in corpus striatum were significantly greater than those of the normothermic controls. Treatment with an i.v. dose of DXM (8 mg/kg) 70 min after the start of heat exposure (or at the time of the onset of heat stroke) significantly attenuates the heat stroke-induced arterial hypotension, cerebral ischemia, and increased levels of glutamate, glycerol, and lactate/pyruvate ratio in corpus striatum.

### 2.3. DXM Attenuates Heat Stroke-Induced Hypercoagulable State

**Figure 2:**
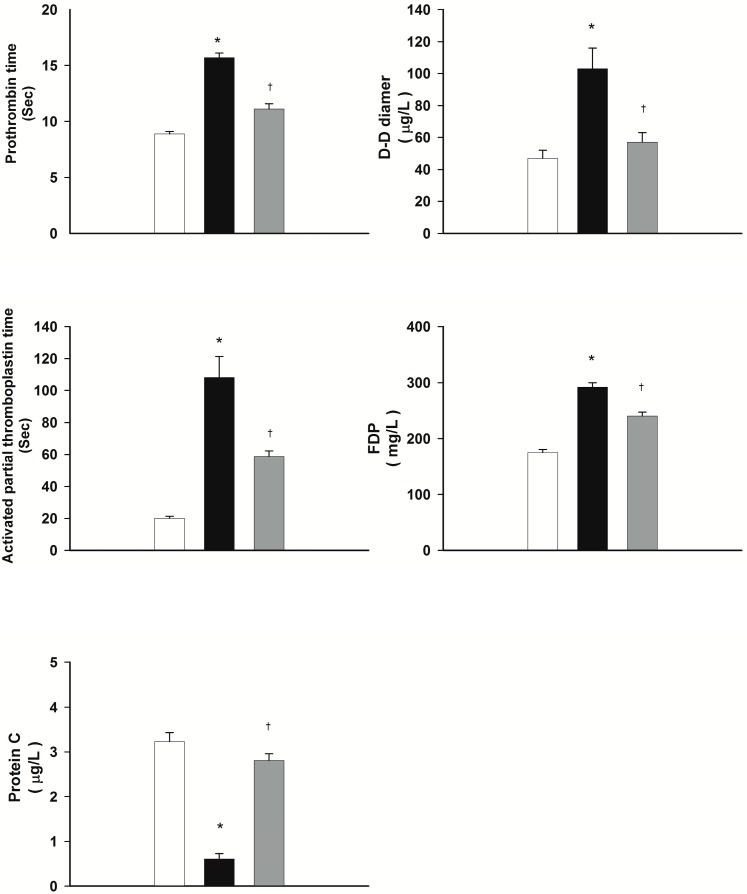
Effects of heat exposure (43 °C) on plasma levels of prothrombin time (PT), activated partial thromboplastin time (aPTT), fibrinogen degradation products (FDP), D-D dimer, and protein C in normothermic control rats (white bar), saline-treated (black bar) or dexamethasone-treated rats (grey bar, 8 mk/kg b.w., i.v.). * *p <* 0.05, in comparison with normothermic control rats; ^†^ *p <* 0.05, in comparison with saline-treated rats (ANOVA followed by Duncan’s test). The values were obtained 85 min after the initiation of heat exposure (or 15 min after the onset of heat stroke) in heat stroke rats or the equivalent time in normothermic controls. Bars are each the mean ± S.E. of 8 rats for each groups.

Figure 2 summarizes the plasma levels of prothrombin time (PT), activated partial thromboplastin time (aPTT), fibrinogen degradation products (FDP), protein C, and D-D dimer for normothermic controls, normal saline-treated heat stroke rats, and DXM-treated heat stroke rats. It can be seen from Figure 2 that PT, aPTT, FDP, and D-D dimer values during heat stroke for rats treated with normal saline (1 mL/kg b.w., i.v.) are all significantly higher at 85 min after the start of heat exposure than those of the normothermic controls. On the contrary, the value for plasma of protein C is significantly lower than that of the normothermic controls. In turn, administration with DXM (8 mg/mL b.w., i.v.) at 70 min after initiation of heat exposure (or immediately at the onset of heat stroke) appreciably attenuates the heat stress-induced increased plasma levels of PT, aPTT, FDP, and D-D dimer as well as the decreased plasma levels of protein C.

### 2.4. DXM Protects from Hepatic and Renal Dysfunction during Heat Stroke

**Figure 3:**
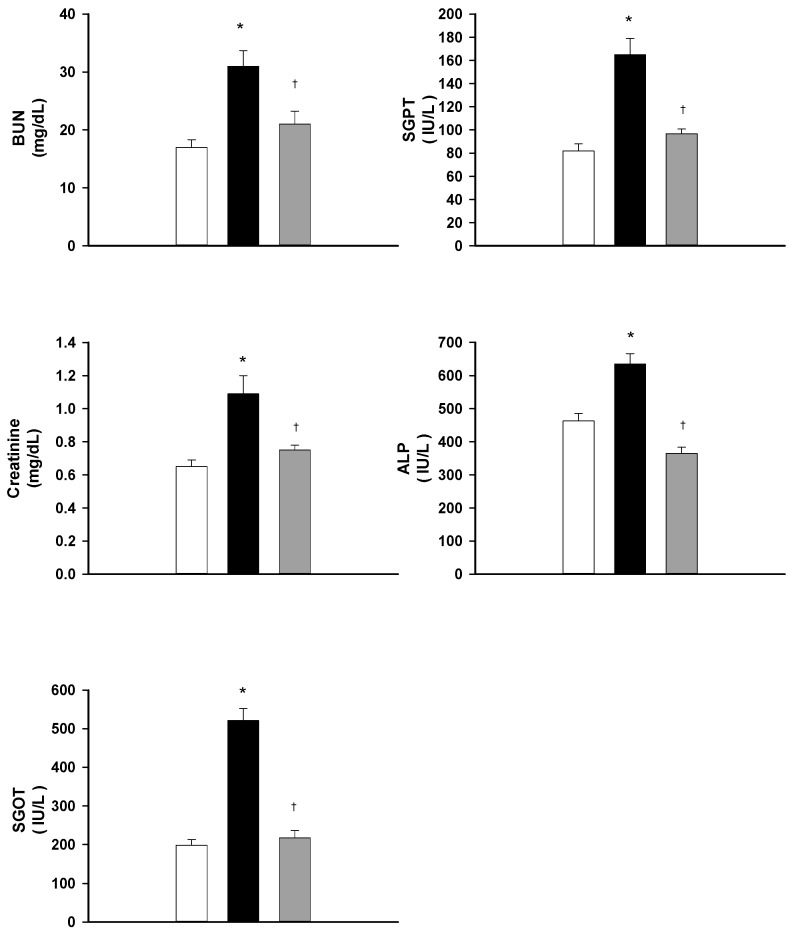
Effects of heat exposure (43 °C) on plasma levels of BUN, creatinine, SGOT, SGPT, and ALP in normothermic control rats (white bar), saline-treated (black bar) or dexamethasone-treated rats (grey bar, 8 mk/kg b.w., i.v.). * *p <* 0.05, in comparison with normothermic control rats; ^†^ *p <* 0.05, in comparison with saline-treated rats (ANOVA followed by Duncan’s test). The values were obtained 85 min after the initiation of heat exposure (or 15 min after the onset of heat stroke) in heat stroke rats or the equivalent time in normothermic controls. Bars are each the mean ± S.E. of 8 rats for each groups.

Plasma levels of blood urea nitrogen (BUN), creatinine, glutamic oxaloacetic transaminase (SGOT), glutamic pyruvic transaminase (SGPT), and alkaline phosphatase (ALP) for normothermic controls, normal saline-treated heat stroke rats, and DXM-treated heat stroke rats are summarized in Figure 3. It can be seen from the figure that the plasma levels of BUN, creatinine, SGOT, SGPT, and ALP for heat stroke rats treated with normal saline (1 mL/kg b.w., i.v.) are significantly higher at 85 min after the start of heat exposure than these are for normothermic controls. Acute treatment with DXM (8 mg/mL b.w., i.v.) at 70 min after initiation of heat exposure (or immediately at the onset of heat stroke) significantly attenuates the heat stress-induced increased plasma levels of BUN, creatinine, SGOT, SGPT, and ALP.

### 2.5. DXM Reduces both IL-1β and Tumor Necrosis Factor-α (TNF-α) Increase but Enhances Interleukin-10 (IL-10) during Heat Stroke

**Figure 4:**
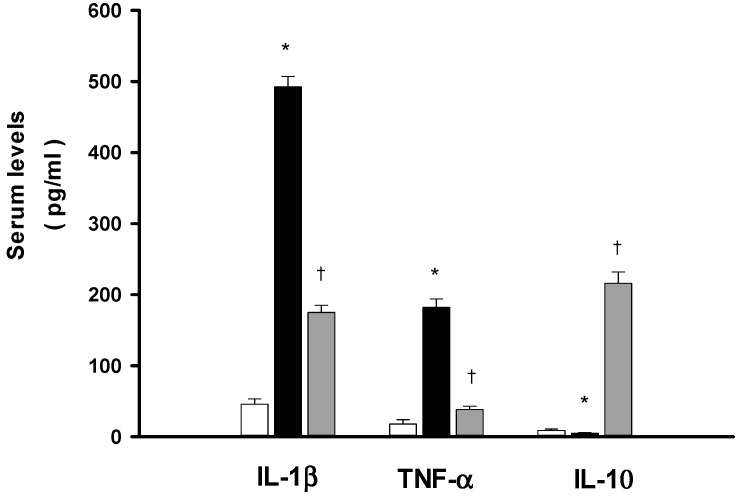
Effects of heat exposure (43 °C) on serum levels of IL-1β, TNF-α and IL-10 in normothermic control rats (white bar), saline-treated (black bar) or dexamethasone-treated rats (grey bar, 8 mk/kg b.w., i.v.). * *p <* 0.05, in comparison with normothermic control rats; ^†^ *p <* 0.05, in comparison with saline-treated rats (ANOVA followed by Duncan’s test). The values were obtained 85 min after the initiation of heat exposure (or 15 min after the onset of heat stroke) in heat stroke rats or the equivalent time in normothermic controls. Bars are each the mean ± S.E. of 8 rats for each groups.

The serum IL-1β and TNF-α, and IL-10 levels for normothermic controls, NS-, and DXM-treated heat stroke rats are summarized in Figure 4. It can be seen from the figure that the serum IL-1β and TNF-α levels in NS-treated heat stroke rats are all significantly higher at 85 min after the start of heat stress than those of the normothermic controls. The acute treatment with DXM (8 mg/mL b.w., i.v.) at 70 min after initiation of heat exposure (or immediately at the onset of heat stroke) significantly attenuates the heat stroke-induced increased serum levels of IL-1β and TNF-α. In NS-treated heat stroke rats, the serum level of IL-10 is maintained at an extremely low level. However, the serum of IL-10 is greatly elevated in heat stroke rats treated with an i.v. dose of DXM (8 mg/mL b.w.).

### 2.6. Discussion

During heat stroke, rodents display hyperthermia, arterial hypotension, intracranial hypertension, cerebral ischaemia, neuronal damage and overproduction of inflammatory cytokines [6,11,12]. The present results, as well as our previous results [3,4,13] revealed that all heat-stressed animals displayed systemic inflammation and activated coagulation, evidenced by increased TNF-α, IL-1β, PT, aPTT, and D-D dimer, and decreased IL-10 and protein C. Biochemical markers evidenced cellular ischemia and injury/dysfunction: plasma levels of BUN, creatinine, SGOT, SGPT, and ALP, and striatal levels of glycerol, glutamate, and lactate/pyruvate ratio, were all elevated during heat stroke. In contrast, the values of mean arterial pressure and striatal levels of local blood flow were all significantly lower during heat stroke. Our present results further demonstrate the circulatory dysfunction, systemic inflammation, hypercoagulable state, and cerebral ischemia and multiple organ dysfunction occurring during heat stroke. However, these heat stroke-induced pathophysiologic changes can all be significantly suppressed by acute treatment with DXM in a dose-dependent manner at the onset of heat stroke.

Our previous results [10] indicated that pretreatment with DXM (4 mg/kg, i.v.) could attenuate the heat stroke-induced damage; however, acute treatment with DXM (4, 6, 8 mg/kg, i.v.) immediately at the onset of heat stroke can apparently increase the survival time in a dose-dependent manner in the present study. This has more meaningful application for emergency treatment in clinics for heat stroke survival. As mentioned above, it was found that rodents share with humans almost the same heat stroke reactions [11,14,15]. These findings demonstrate that rodent heat stroke models can nearly mirror the full spectrum of human heat stroke. Experimental heat stroke fulfills the empirical triad used for the diagnosis of classical human heat stroke [15,16]. After onset of heat stroke, rats revealed ischemia and injury in several cerebral regions, especially in the corpus striatum. Both of our previous [3,13] and present results displayed that the local CBF of striatum decreased significantly, but the values of striatal neuronal ischemic and injury index were sharply increased in rats with heat stroke (as shown in Figure 1). However, acute immediate treatment with DXM revealed appreciable decrements of neuronal damage, ischemic and hypoxic indexes in rats of heat stroke.

In addition, heat stroke rats showed hepatic failure (evidenced by levels of SGOT, SGPT and alkaline phosphatase) and renal failure (evidenced by increased plasma levels of BUN and creatinine) and hypercoagulable state [17,18,19]. Kew *et al.* [20,21] indicated that hepatic and renal failure may be related to tissue ischemia (due to circulatory shock) and thermal injury. As shown in the present results, heat stroke rats indeed displayed vital organ failure, and these pathophysiological changes were consistent with our previous studies [12,13]. Nevertheless, immediate administration of DXM greatly diminished the severity of cerebral, hepatic and renal failure in rats of heat stroke. So far, there has been no study that tried to investigate the effects of glucocorticoids on blood coagulation state, and hepatic and renal functions in rats during heat stroke. The present study has been focused on whether acute treatment with DXM can improve the heat stroke-induced hypercoagulable state and vital organ dysfunctions, and positive and meaningful findings were obtained.

The serum concentrations of inflammatory cytokines including TNF-α and IL-1β are overproduced in human victims and in rodents with heat stroke [4,22,23]. Evidence has demonstrated that expression of cytokines correlates well with the severity of heat stroke [23,24]. The hypotension, intracranial hypertension and cerebral ischemia that occurred during heat stroke can be mimicked by intravenous administration of infusion of IL-1β [25], but prevented by prior antagonism of IL-1β receptors [26,27]. Indeed, our previous studies have also shown that heat stroke induces systemic overproduction of TNF-α and IL-1β in rodents [3,4], as well as shown in the present results that an increase of serum IL-1β and TNF-α levels is observed in heat stroke rats. Furthermore, the present study shows that acute immediate treatment with DXM diminishes the heat stroke-induced elevation in serum levels of TNF-α and IL-1β. Meanwhile, both arterial hypotension and cerebral ischemic damage are prevented and survival of heat stroke rats is improved following acute immediate administration of DXM at the onset of heat stroke. The present results further indicate that acute treatment with DXM causes a significant increase in the serum level of IL-10 during heat stroke. Studies found that IL-10 has important anti-inflammatory and immunosuppressive properties through attenuation of proinflammatory cytokines [28,29]. In our present study, acute immediate administration of DXM may improve arterial hypotension and cerebral ischemia and prevent damage by increasing IL-10 but suppressing levels of TNF-α and IL-1β.

It has been shown that the glutamate and lactate/pyruvate ratio are well-known markers of cellular ischemia, whereas glycerol is a marker of how severely cells are affected by ongoing pathology [30,31,32,33,34,35]. Indeed, as shown in our previous [3,4] and present results, cerebral ischemia induced by heat stroke is associated with an increased production of glycerol, lactate/pyruvate ratio and glutamate in the brain as well as a decreased level of MAP in the periphery. It has been reported [36] that the increased glutamate in the brain during the rat heat stroke also mediated the development of neuronal damage. Cerebral glutamate overload resulting from arterial hypotension and intracranial hypertension might be responsible for the occurrence of central nervous system syndromes associated with heat stroke [36,37]. Systemic administration of glutamate receptor antagonists could protect against ischemic neuronal injury in experimental heat stroke [36,37,38]. In addition, recent studies reveal the excessive accumulation of cytotoxic free radicals in the brain and oxidative stress occurred during heat stroke [4,39]. Evidence had accumulated to suggest that heat stroke-induced cerebral ischemia and neuronal damage might be associated with an increased production of free radicals [4]. Pretreatment with hydroxyl radicals scavengers, such as α-tocopherol, prevented production of hydroxyl radicals, reduced lipid peroxidation and ischemic neuronal damage in several brain areas (corpus striatum, hypothalamus and cortex) of rats exposed to heat stroke and prolonged subsequent survival [40]. After the onset of heat stroke, the cessation or reduction of blood flow to the brain induced neuronal damage. This neurotoxic cascade involved overproduction of glutamate in the brain [4,36,37]. In this study, our findings have indicated that the improvement of animal survival and the cerebral neuronal damage by acute DXM administration are also associated with the amelioration of cerebral glutamate, suggesting that DXM may exert its neuroprotective effect via attenuation of glutamate overload. To delineate whether the DMX effect also involves the removal of other deterioration factors or neurotoxins (e.g., oxidative substances, lipid peroxidation products), further investigations into the precise mechanisms of DXM-mediated neuroprotective effect will be required.

The current choice for treatment of heat stroke is immediate administration of DXM in a dose dependent manner. However, DXM efficacy was tested in a baboon model of heat stroke, despite dexamethasone treatment prior to heat stress and during cooling, protection against the lethal effects of heat stroke were not realized [41]. Similarly, the treatment of synthetic GC for human and animal-spinal cord injury or cerebral ischemia has been shown to be beneficial [8,9], but some studies have demonstrated that administration of steroids do not improve mortality or mobility after an acute ischemic brain stroke or in acute spinal cord injury [42,43]. Xue *et al.* [44] indicated that perhaps short-term and high dose of GCs are cardioprotective, albeit long-term and inadequate dose of glucocorticoid exposure may cause quite different consequences of deleterious cardiac function. In the aspect of heat stroke, although DXM caused sustained elevation of plasma interleukin-6 levels and decreased complement system activation with similar mortality rates between control and treated animals in a baboon model of heat stroke [41], our present findings suggest that rats treated with the synthetic GC, DXM, show improved heat stroke tolerance, as illustrated by attenuation of hypotension, cerebral ischemia, and neuronal damage and a prolongation to survival time. Perhaps GC efficacy is thought to be at least partially dependent on appropriate dosage or heat severity, as metyrapone (an inhibitor of corticosterone synthesis) is without effect on cytokine mRNA expression, except at high heat loads in which it induces increased TNF-α mRNA expression [45]. Clearly, whether permissive actions of GCs are sufficient for cytokine regulation and heat stroke protection or stress-induced levels are required is currently unknown, but the mechanism of protection of DXM in the present study appears to be at least partially mediated through the inhibition of IL-1-β TNF-α, but not promotion of IL-10 actions. Furthermore, more studies are required in this area to discuss and determine the potential benefit of GC therapy as a heat stroke prevention and treatment strategy.

## 3. Materials and Methods

### 3.1. Experimental Animals

Adult male Sprague-Dawley rats weighing between 280 and 330 g were obtained from the Animal Resource Center, National Science Council of Republic of China (Taipei, Taiwan, ROC). Between experiments the animals were housed individually at an ambient temperature of 24 ± 1 °C with a 12 h light-dark cycle, with the lights being switched on at 0600 h. Animal chow and water were allowed ad libitum. All experiments were approved by the Animal Ethics Committee of the Chia-Nan University of Pharmacy and Science, Tainan, Taiwan (approbated No. CN-IACUC-96032). Animal care and experiments were conducted according to the Guide for the Care and Use of Laboratory Animals of the National Institutes of Health and the guidelines of the Animal Welfare Act.

### 3.2. Surgery and Physiological Parameter Monitoring

The femoral artery and vein of rats, under urethane anesthesia, were cannulated with polyethylene tubing (PE50) for blood pressure monitoring from the right femoral artery, blood samplings (for cytokines assay from right femoral vein, and for biochemical measurements from left femoral artery) and drug administration into left femoral vein. The animals were positioned in a stereotaxic apparatus (Kopf model 1460, Grass. Instrument. Quincy, MA, USA) to allow insertion of probes for measurement of CBF. Physiological monitorings included Tco, MAP, HR, and CBF in the corpus striatum.

### 3.3. Induction of Heat Stroke and Experimental Design

Rats under anesthesia were randomly assigned to one of the following five groups. One group of rats was exposed to an ambient temperature (Ta) of 24 °C for at least 90 min to reach thermal equilibrium before they were tested and used as normothermic controls. They were treated with 0.9% NaCl solution (1 mL/kg, i.v.) at 70 min after the start of the experiments. Their Tco was maintained at about 36 °C using an electric thermal mat before the start of experiments. The second group of rats with heat stroke received 0.9% NaCl solution (1 mL/kg, i.v.) 70 min after heat exposure. Heat stroke was induced by exposing the animals to an ambient temperature of 43 °C (with a relative humidity of 60% in a temperature-controlled chamber). The instant at which MAP and local CBF began to sharply decrease from their peak levels was taken as the onset of heat stroke [3], as shown in Figure 1. Our pilot results displayed that the interval between the start of heat exposure and onset of heat stroke was found to be 70 ± 3 min (*n* = 8). Accordingly, in the following heat stroke groups, all rats were exposed to 43 °C for exactly 70 min and after the onset of heat stroke, and allowed to recover at room temperature (24 °C). The other three groups of rats with heat stroke respectively received DXM 4, 6, and 8 mg/mL/kg i.v. at the onset of heat stroke (70 min after the start of heat exposure). Each group of animals was subjected to: (i) measurement of survival time; (ii) measurement of Tco, MAP, HR, CBF; and striatal concentration of glutamate, glycerol, and lactate/pyruvate; (iii) measurement of plasma levels of PT, aPTT, protein C, D-D dimer, and FDP; (iv) measurement of plasma levels of BUN, creatinine, SGOT, SGPT and ALP; (v) measurement of serum levels of interleukin-1β (IL-1β), TNF-α, and IL-10. Colon temperature was monitored continuously by a thermocouple, while MAP and HR were monitored with a pressure transducer. Adequate anesthesia was maintained to abolish the corneal reflex and pain reflexes induced by tail pinch throughout the course of all experiments (about 8 h) following a single dose of urethane (1.4 g/kg b.w., i.p.). At the end of the experiments, control rats were killed with an overdose of urethane.

### 3.4. Measurement of CBF

Local CBF in the corpus striatum (SBF) was monitored with a Laserflo BPM2 laser Doppler flowmeter (Vasametics, St. Paul, NM, USA). A 24 gauge stainless steel needle probe (diameter, 0.58 mm; length, 40 mm) was inserted into the right corpus striatum using the coordinates: A, interaural 9.7 mm; L, 2.0 mm from mid-line; and H, 4.5 mm from the top of the skull [41].

### 3.5. Measurements of Extracellular Ischemia and Damage Markers in Brain

After cannulation of vessels, the animal’s head was mounted on a stereotaxic apparatus with the nose bar positioned 3.3 mm below the horizontal line. Following a midline incision, the skull was exposed and a burr hole was made in the skull for the insertion of a dialysis probe (4 mm in length, CMA/12, Carnegie Medicine, Stockholm, Sweden). The microdialysis probe was stereotaxically implanted into the corpus striatum according to the atlas and coordinates of Paxinos and the coordinates of Paxinos and Watson (1982) [46]. As the methods described previously, [4,13] an equilibrium period of 2 h without sampling was allowed after probe implantation. The dialysis probe was perfused with Ringer’s solution (147 mM Na^+^, 2.2 mM Ca^2+^, 4 mM K^+^, pH 7.0) at 2 μL/min using a CMA/100 microinfusion pump. Dialysates were collected every 10 or 20 min in a CMA140 fraction collector. Aliquots of dialysates (5 μL) were injected onto a CMA600 Microdialysis Analyzer (Carnegie Medicine) for measurement of lactate, glycerol, pyruvate and glutamate. Four analytes can be analyzed per sample and the result is displayed graphically within minutes. The thermal experiments were started after showing stabilization in four consecutive samples.

The lactate/pyruvate ratio is a well-known marker of cell ischemia, that is, an inadequate supply of oxygen and glucose [30,31]. Glycerol is a marker of how severely cells are affected by the ongoing pathology [32,33]. Glutamate is released from neurons during ischemia and initiates a pathological influx of calcium leading to cell damage. It is an indirect marker of cell damage in the brain [34,35].

### 3.6. Biochemical Measurements

For biochemical determination, blood samples at 85 min after the start of heat exposure (or 15 min after the onset of heat stroke) were drawn by arterial femoral cannulation. The plasma levels of activated partial thromboplastin time, prothrombin time, and D-D dimer were measured by automated coagulation instruments (SYSMEX CA-1500, Kobe, Japan). The plasma levels of SGOT, SGPT, and alkaline phosphatase were determined by spectrophotometry (HITACHI 7600, Tokyo, Japan). For determination of plasma protein C, plasma was prepared as described previously [13], protein C in the sample was activated by specific venom activator. The resulting protein C activator was assayed in a kinetic test by measuring the increase in absorbance at 405 nm. The reagents for the determination of protein C activity were provided by Berichrom Protein C (Dade Behring Marburg GmbH, Marburg, Germany).

### 3.7. Measurement for Serum Cytokines

Blood samples were taken at 85 min after the start of heat exposure (or 15 min after the onset of heat stroke) for determination of IL-1β, TNF-α, and IL-10 levels. For measurement of serum cytokines, 5 mL of blood was drawn from the femoral vein of rats. The amounts of the cytokines including IL-1β, TNF-α, and IL-10 in serum were determined by using a double-antibody sandwich enzyme-linked immunoabsorbant assay (ELISA, R&D Systems, Minneapolis, MN, USA) according to the manufacturer’s instructions. This assay employs the quantitative colorimetric sandwich ELISA technique. Optical densities were read on a plate reader set at 450 nm for IL-1β, TNF-α, and IL-10. The concentration of these cytokines in the serum samples was calculated from the standard curve multiplied by the dilution factor and was expressed as pg/mL.

### 3.8. Data Analysis

Data are presented as means ± S.E. Repeated-measures ANOVA is conducted to test the treatment by time interactions and the effect of treatment over time on each score. The Duncan multiple-range test is used for post hoc multiple comparisons among means. A *p-*value less than 0.05 is calculated as statistical significance.

## 4. Conclusions

In summary, the prolongation of survival time in rats with immediate DXM therapy adopted at the onset of heat stroke was found to be associated with augmentation of both arterial blood pressure and cerebral blood flow, as well as reduction of cerebral ischemia, and vital organ damage, activated coagulation, and systemic inflammation during heat stroke. Our present results have shown a convincingly significant dose-dependent therapeutic effect of DXM administered immediately at the onset of heat stroke. Altogether, our data support that DXM may exert its therapeutic benefits by suppressing both cytokine overproduction and hypercoagulable state during heat stroke.
